# Powder metallurgy as a perfect technique for preparation of Cu–TiO_2_ composite by identifying their microstructure and optical properties

**DOI:** 10.1038/s41598-023-33999-y

**Published:** 2023-04-29

**Authors:** Ashraf K. Eessaa, Omayma A. Elkady, A. M. El-Shamy

**Affiliations:** 1 Nanotechnology central lab, Electronics Research Institute (ERI), Cairo, Egypt; 2 Powder Technology Department, Center Metallurgical Research & Development Institute (CMRDI), Cairo, Egypt; 3 Physical Chemistry Department, Electrochemistry and Corrosion Lab., National Research Centre, El-Bohouth St. 33, Dokki, P. O. 12622, Giza, Egypt

**Keywords:** Engineering, Materials science, Nanoscience and technology

## Abstract

Powder metallurgy (PM) is a technique that involves the manufacturing of metal powders and their consolidation into finished products or components. This process involves the mixing of metal powders with other materials such as ceramics or polymers, followed by the application of heat and pressure to produce a solid, dense material. The use of PM has several advantages over traditional manufacturing techniques, including the ability to create complex shapes and the production of materials with improved properties. Cu–TiO_2_ composite materials are of great interest due to their unique properties, such as high electrical conductivity, improved mechanical strength, and enhanced catalytic activity. The synthesis of Cu–TiO_2_ composites using the PM technique has been gaining popularity in recent years due to its simplicity, cost-effectiveness, and ability to produce materials with excellent homogeneity. The novelty of using the PM technique for the preparation of Cu–TiO_2_ composite lies in the fact that it enables the production of materials with controlled microstructures and optical properties. The microstructure of the composite can be fine-tuned by controlling the particle size and distribution of the starting powders, as well as the processing parameters such as temperature, pressure, and sintering time. The optical properties of the composite can also be tailored by adjusting the size and distribution of the TiO_2_ particles, which can be used to control the absorption and scattering of light. This makes Cu–TiO_2_ composites particularly useful for applications such as photocatalysis and solar energy conversion. In summary, the use of Powder Metallurgy for the preparation of Cu–TiO_2_ composite is a novel and effective technique for producing materials with controlled microstructures and optical properties. The unique properties of Cu–TiO_2_ composites make them attractive for a wide range of applications in various fields, including energy, catalysis, and electronics.

## Introduction

Powder metallurgy is a versatile and widely used technique for producing composite materials. In recent years, the preparation of Cu–TiO_2_ composites using powder metallurgy has gained significant attention due to its potential applications in various fields such as aerospace, electrical, and biomedical industries^1^. The main advantages of using this technique for the preparation of Cu–TiO_2_ composites include its ability to control the microstructure of the composite, its low cost, and its high efficiency. In this essay, we will discuss the innovative and research value of powder metallurgy as a perfect technique for the preparation of Cu–TiO_2_ composite by identifying their microstructure and optical properties^2^.

The first innovative aspect of powder metallurgy is its ability to control the microstructure of the composite material^3^. Powder metallurgy involves the mixing of metal powders with ceramic particles, which are then compacted and sintered to produce the final composite. The process allows for the precise control of the particle size, distribution, and orientation of the ceramic particles in the metal matrix^4^. This enables the optimization of the mechanical, electrical, and optical properties of the composite material. In the case of Cu–TiO_2_ composite, the microstructure of the composite can be tailored to achieve desirable properties such as high hardness, high wear resistance, and good electrical conductivity^5^.

The second innovative aspect of powder metallurgy is its low cost. Compared to other techniques such as casting or forging, powder metallurgy is a more cost-effective method for producing composite materials^6^. This is because the process allows for the efficient use of raw materials, with minimal waste. Additionally, the technique is highly automated, which reduces labor costs and improves the reproducibility of the final product^7^.

The third innovative aspect of powder metallurgy is its high efficiency. The technique enables the production of complex shapes and geometries, which is not possible with other methods such as casting or forging^8^. This is because the process involves the use of molds and dies, which can be easily designed to produce complex shapes. In the case of Cu–TiO_2_ composite, the technique can be used to produce components with intricate shapes and features, which is useful for applications such as microelectronics and medical implants^9^.

The microstructure of Cu–TiO_2_ composite produced by powder metallurgy is another important aspect that contributes to its innovative and research value. The microstructure of the composite material determines its mechanical, electrical, and optical properties^10^. In the case of Cu–TiO_2_ composite, the microstructure can be tailored to achieve desirable properties such as high hardness, wear resistance, and good electrical conductivity^11^. The microstructure of the composite can be analyzed using various techniques such as scanning electron microscopy (SEM) and X-ray diffraction (XRD).

The optical properties of Cu–TiO_2_ composite are another important aspect that contributes to its innovative and research value^12^. The composite exhibits excellent optical properties due to the presence of TiO_2_ particles in the metal matrix. TiO_2_ is a well-known photocatalyst, and its incorporation into the metal matrix results in a material that exhibits excellent optical properties such as high transparency and excellent UV-absorption^13^. These properties make the composite material useful for applications such as solar cells, sensors, and optical coatings^14^.

The innovative and research value of powder metallurgy as a perfect technique for the preparation of Cu–TiO_2_ composite can also be seen in its applications in various fields such as aerospace, electrical, and biomedical industries^15^. In the aerospace industry, the composite material can be used to produce components such as turbine blades, which require high strength and wear resistance^16^. In the electrical industry, the composite material can be used to produce electrical contacts, which require good electrical conductivity and wear resistance. In the biomedical industry, the composite material can be used to produce medical implants^17^.

The study aims to enhance the photocatalytic activity of copper nanoparticles for various applications^18^. Copper is considered one of the best metals to be used in conjunction with titanium dioxide (TiO_2_) surfaces, as it can significantly increase the amplification of photocatalytic activity. To achieve this, TiO_2_-doped copper nano catalysts were created through mechanical milling^19^. The study utilized different weight percentages of titanium dioxide, and stearic acid was used as a process control agent^20^. The researchers found that Cu–TiO_2_ powder nanocomposites with 10, 20, 30, and 40 weight percent of titanium dioxide exhibited high photocatalytic activity^21^. The study also mentioned the use of TiO_2_ in various everyday products, such as paints, papers, hydrogen gas evolution, and cosmetic goods, among others. Furthermore, the article discussed the advantages of using titanium dioxide as a semiconductor nanomaterial in photocatalytic applications due to its optimal optical and electron characteristics, corrosion resistance, chemical stability, and non-toxicity^22^. Despite its broad bandgap, TiO_2_ is a popular choice for use as a buffer layer in solar cells and can be improved through various techniques such as creating composite materials and doping with suitable metal atoms. Copper is a promising dopant for TiO_2_, as it has better electrical conductivity and is more readily available and less expensive than other metals like silver^23^.

## Materials and methods

### Materials

In this paper, pure copper was selected as a matrix material as it has been generally utilized in many recent applications. Titanium dioxide was chosen to be used as a reinforcement material to manufacture the samples. A Copper powder with 99.9% purity (supplied by Alpha Chemicals, USA) with a 10 μm average particle size has been used as a metal matrix. TiO_2_ powder with 99.7% purity (supplied by Alpha Chemicals, USA) with an average size of 50 nm has been used as reinforcement. The average size of Cu and TiO_2_ powder in Fig. 4a and b is much larger than 10 μm and 50 nm, respectively, mentioned in the part of raw materials. The data source of the average particle size was supplied by Alpha Chemicals, USA.

### Preparation of Cu–TiO_2_ photocatalysts

**Figure 1:**
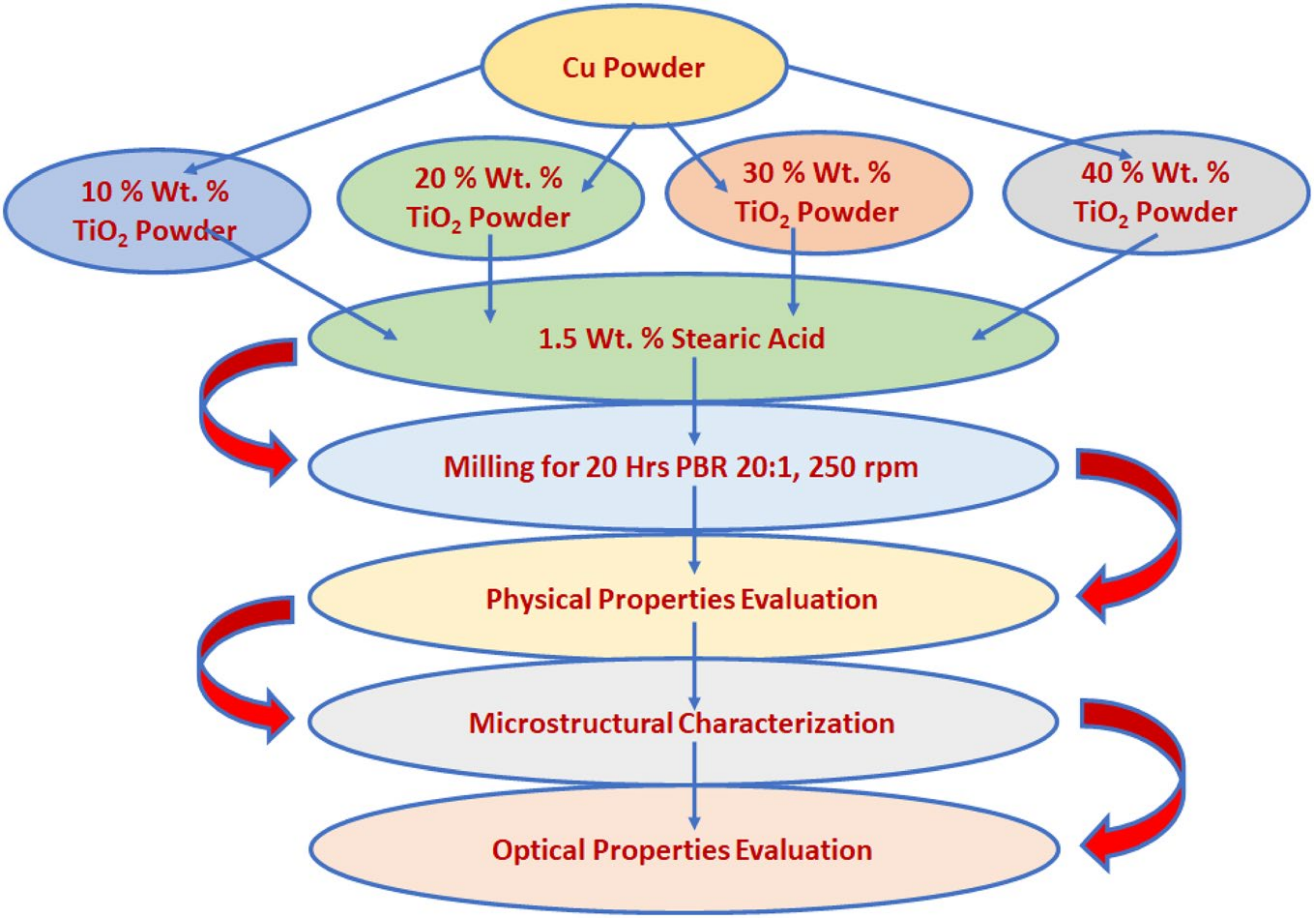
Schematic diagram for fabrication of Cu–TiO_2_ composite powders at a different weight percent of TiO_2_ 10, 20, 30, and 40 wt.%.

Cu powder mixtures contain 10, 20, 30 & 40 wt.% TiO_2_ which has been mixed using Zirconia ceramic balls is used in the mechanical mixing process, in which if stainless steel balls are used some contaminations with iron can to takes place. But Zirconia balls are inert for any reaction and so hard. The ball mill used in the preparation process in planetary four vails ball mill machine. The copper powder used in this work is atomized semispherical copper. A ball mill technique for 24 h until a homogeneous mixture is obtained. Table 1 summarizes the specifications of the matrix and reinforcements employed for this study. This paper applied the powder metallurgy method to produce the recommended hybrid Cu–TiO_2_ nanocomposite. First, the composite powders of Cu and TiO_2_ were weighed concerning the required fractions using a sensitive electronic balance of 0.1 mg accuracy level. Then, the weighted composite powders were mixed in a stainless-steel vial and protected from oxidation using pure argon, with a 20:1 steel ball-to-powder ratio (BPR), a ball diameter of 5 mm, and a rotational speed of 250 rpm. Stearic acid (1.5 wt.%) was used as a process-controlling agent (PCA). Figure 1 shows the composition and nomenclature of prepared specimens.

**Table 1:** Representative the composition and nomenclature of prepared specimens.

Serial	Code	Composition	
		Reinforcement	Matrix
		TiO_2_	Cu
1	S1	90 ± 1.0%	10 ± 1.0%
2	S2	80 ± 1.0%	20 ± 1.0%
3	S3	70 ± 1.0%	30 ± 1.0%
4	S4	60 ± 1.0%	40 ± 1.0%

### Characterization of Cu–TiO_2_ composite for the photocatalysts

The investigations on the microstructural characteristics of the composite powders were achieved using scanning electron microscopy and energy-dispersive spectroscopy (SEM/EDS). The main goal of using such analysis techniques is to identify the uniform dispersal of the reinforcement materials in the matrix, the composites microstructure, and the composites phases. According to that, this paper also applied the X-ray diffraction (XRD) technique to identify the phases of the mixed powders using a diffractometer with Cu K-alpha radiation and operated at 40 kV. The samples are examined using IR spectroscopy to investigate the absorption band spectra. Also, the antireflection properties were studied.

On the other hand, the electrical and thermal conductivities were evaluated using the PCE-COM20 electric resistivity instrument. Thermal conductivity can be calculated using Eq. (1)^24^.

1 $$\mathrm{K }=\mathrm{ LT\sigma },$$

where K refers to the thermal conductivity in W/m. K, L is the Lorentz number (for composites L = 2.45 × 10^–8^ W Ω K^−2^), T denotes the absolute temperature in K, and finally, σ is the electrical conductivity in Ω^−1^ m^−1^.

### Conductivity of the composite powder

Samples for the electrical conductivity measurements were produced by compacting the milled powders at a pressure of 0.37 GPa at 90C. The diameter of the samples was 10 mm with a height of 6 mm. The electrical resistivity of the compacted samples was measured at room temperature (50% relative humidity) between gold electrodes with an alternating current method at a frequency of 1 kHz.

## Results and discussion

### XRD analysis

The main task of XRD is to determine and examine the phase composition & phase structure of Cu–TiO_2_ crystallinity. XRD is a non-destructive method that is widely used to characterize crystalline material. The structure, phase, crystallinity, and materials' sizes have been shown by using XRD analysis. Scherrer equations are used to calculate the crystal size of the material^25^.

2 $$\mathrm{d}=\frac{\mathrm{k \lambda }}{\mathrm{\beta cos\theta }},$$

where d is crystallite size, β is the full width of half maximum, θ is the diffraction angle, and λ is the X-ray wavelength of X-ray radiation^26^.

**Figure 2:**
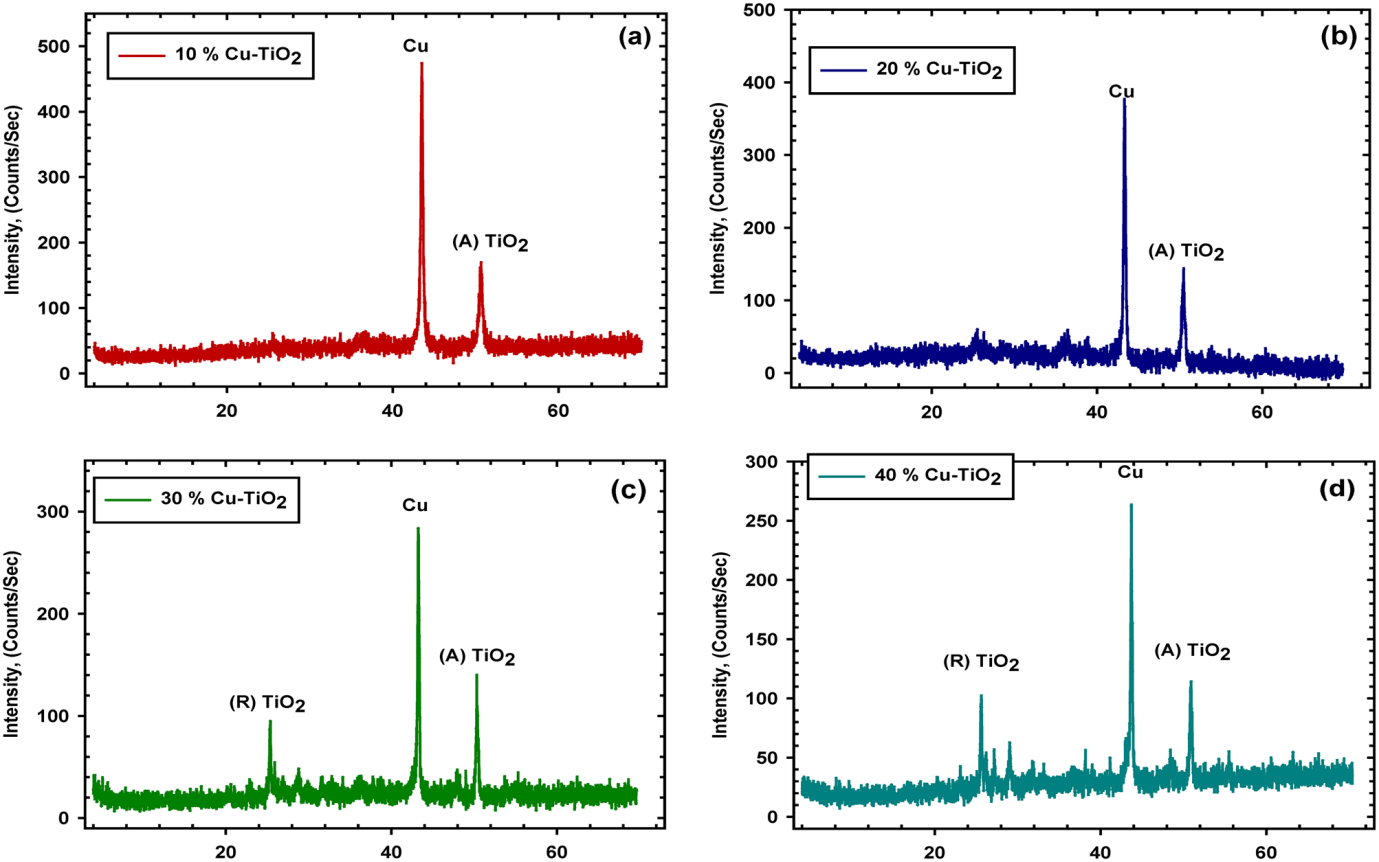
Representative the XRD patterns of various TiO_2_ concentrations doped in Cu matrix (**a**) 10, (**b**) 20, (**c**) 30 and (**d**) 40 wt.% of TiO_2_.

The diffraction patterns for various concentrations of TiO_2_ doped in Cu are shown in Fig. 2. Two phases of tetragonal TiO_2_ are seen when doped with 40% TiO_2_; one of these phases is anatase TiO_2_ (the peak with the highest intensity), and the other phase is rutile TiO_2_ (the peak with the lowest intensity that is adjacent to the peak with the highest intensity, which represents a very small amount of rutile TiO_2_) (the as-resaved TiO_2_ powder has both the anatase & rutile phases). High photocatalytic activity may be attributed to this very modest quantity, which functions in the anatase phase as a structural defect or impurity. Following a reduction in the percentage of TiO_2_, only anatase diffraction peaks were seen in samples containing various amounts of TiO_2_. It is also possible to notice that the majority of the 2 peak locations of the primary diffraction pattern do not move, having identical values of pure Cu, except for variations in the intensities of these peaks. This is something that can be seen in all the samples (i.e. intensity decreases as TiO_2_ increases). Because the radii of Ti^4+^ ions are too large to replace Cu^+^ ions in the Cu matrix, the addition of TiO_2_ did not result in any significant modifications to the crystallinity of the material. Aside from the peaks associated with copper and titanium dioxide, there are no other peaks that relate to any novel compounds or phases. This is a reference to the lack of a reaction between copper and titanium dioxide.

There have been no observations of any intermetallic peaks between copper and titanium dioxide, and this is a direct result of the well-regulated milling done by the machine. The structure of the copper metal is known as FCC (Face Centered Cubic), and its atomic radius is 128 pm. In contrast, the structure of titanium is known as HCP (Hexagonal Close Packed), and its atomic radius is 147 pm. Cu with its smaller atomic radius can replace Ti atoms or interstitial incorporated in the Ti crystals, which is caused by the interaction between the crystals of Cu and Ti, which is caused by the mixing of Cu and TiO_2_ using mechanical milling for a long time with a high rotation speed. This happens because of the interaction between the crystals of Cu and Ti. Because of this, there are certain shifts in the crystallite structure, which is a sign of successful mixing between the Cu matrix and TiO_2_ as a reinforcement. To a greater extent, the interaction between Cu and TiO_2_ may be seen to have taken place as a result of the examination of the crystal structure if one uses appropriate settings for the mechanical milling process. To determine the size of the crystallites, the full width at half maximum (FWHM) of the diffraction peaks was used in conjunction with Scherer’s approach. The results of the calculations are given in Table 2. The smaller particle size of the TiO_2_ may lead to a larger specific surface area and the surface-to-volume ratio of the solar cell, as well as an increased bandgap, which can increase the efficiency of the solar cell. Several investigations and measurements have led researchers to the conclusion that reducing the crystalline size of cells based on TiO_2_ can help to improve their photovoltaic output by increasing electron lifetime, facilitating faster electron transport, increasing charge collection efficiency, and reducing the amount of recombination that occurs^27^.

**Table 2:** Representative the values of crystalline size for different TiO_2_ concentrations 10, 20, 30 and 40 wt.%.

Samples	Crystalline size in nm
10% Cu–TiO_2_	46.365
20% Cu–TiO_2_	69.507
30% Cu–TiO_2_	65.658
40% Cu–TiO_2_	111.277

**Figure 3:**
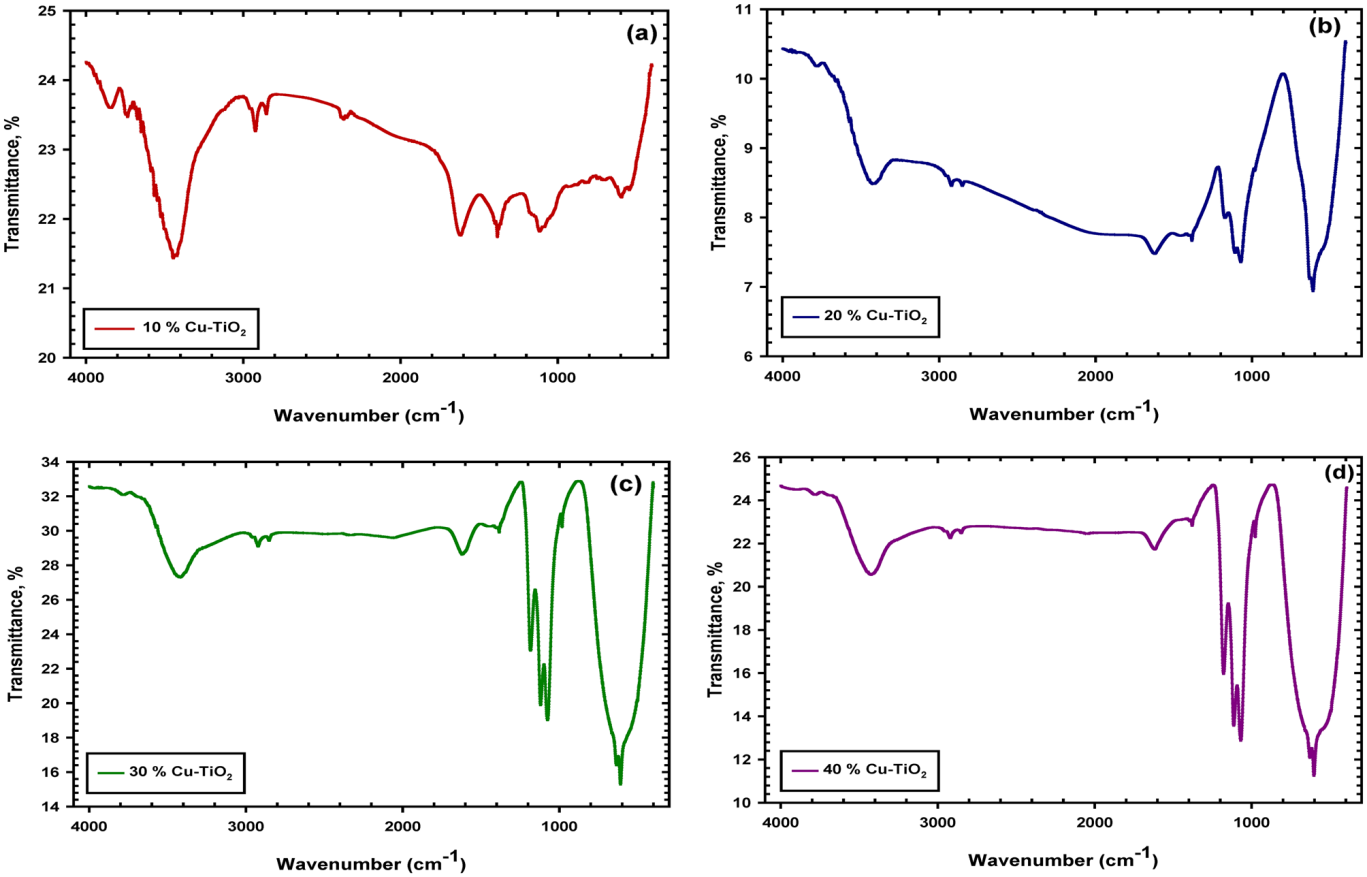
Representative the DRIFTS spectra of Cu–TiO_2_ catalyst at various Cu contents 10, 20, 30, and 40 wt.% of TiO_2_. (**a**) Represents Cu + 10 wt.% of TiO_2_; (**b**) Represents Cu + 20 wt.% of TiO_2_; (**c**) Represents Cu + 30 wt.% of TiO_2_; and (**d**) Represents Cu + 40 wt.% of TiO_2_. The peaks refer to absorption bands in spectra, since at 3426 and 1620 cm^−1^ which are due to OH-stretching and bending vibrations. The Ti–O band was detected between 500 and 900 cm^−1^. The mixed oxide (Ti–O–Cu) bonding appeared at 2922 cm^−1^.

As can be seen in Fig. 3, the OH-stretching and bending vibrations are responsible for the absorption bands in the spectra, which were found at 3426 and 1620 cm^−1^ respectively. Additionally, between 500 and 900 cm^−1^, a band of Ti–O was detected. The intensities of the OH bands (both stretching and bending) and the Ti–O bands, on the other hand, dropped as the amount of Cu in the sample increased^28^. The fact that the peaks in the area between 500 and 1000 cm^−1^ are distinct from those of pure CuO and pure TiO_2_ implies the formation of new metal–oxygen bonds. These findings provide credence to the hypothesis that mixed oxide (Ti–O–Cu) bonding was formed since evidence of this kind of bonding was seen at 2922 cm^−1^. After being treated, it was found that the TiO^2^ photocatalyst had a significant quantity of water vapor and surface hydroxyl groups adsorbed to it^29^.

### Microstructure analysis

**Figure 4:**
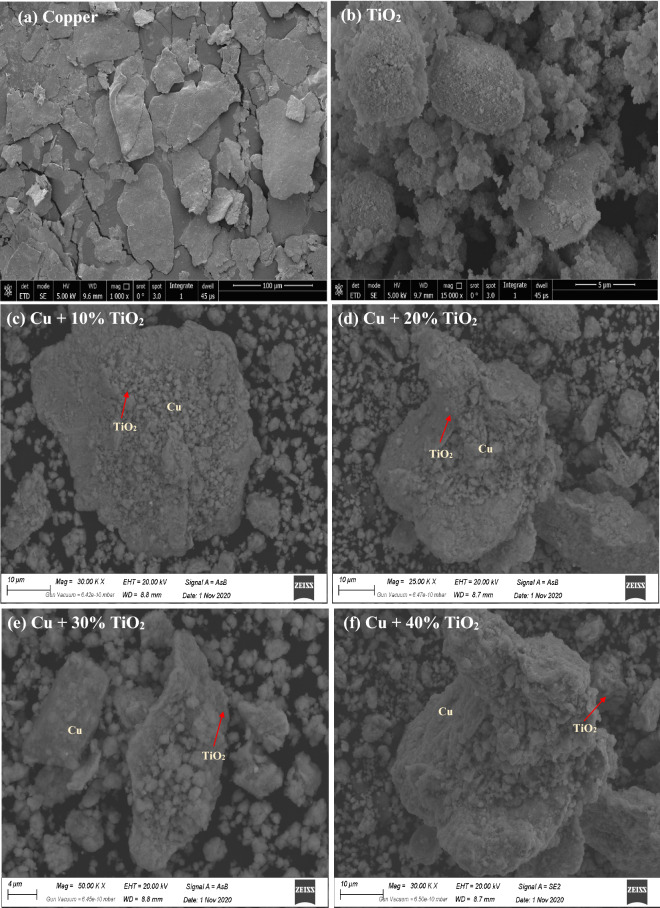
Representative the SEM micrographs of the Cu composite with different concentrations of nano-TiO_2_ (**a**) represents Cu only (**b**) represents Cu only at higher magnification (**c**) represents Cu with 10 wt.% TiO_2_ (**d**) represents Cu with 20 wt.% TiO_2_ (**e**) represents Cu with 30 wt.% TiO_2_ (**f**) represents Cu with 40 wt.% TiO_2_.

SEM micrographs of the Cu composite reinforced with nano-TiO_2_ were illustrated in Fig. 4. Figure 4a and b show the pure Cu, and pure TiO_2_, respectively. The microstructure of Cu–TiO_2_ composite powder, in Fig. 4c–f are corresponding to 10, 20, 30, and 40 wt.% TiO_2_ in the Cu matrix, respectively. As known, the necessary parameter in the production of nanocomposites is proper and well-dispersal nano reinforcement in the metal matrix. The nano-TiO_2_ particles were distributed in an even manner throughout the Cu matrix composite, as shown in Fig. 4. A further observation that can be made is that the nano-TiO_2_ reinforcing material was dispersed appropriately, entrapped inside the Cu matrix, and adhered very strongly since TiO_2_ is a substance that is useful for the operation of solar cells. As a result, enhancing the photocatalytic activity of the created Cu-TiO_2_ composite by adding it to the copper matrix at a nanoscale in amounts of a considerable percentage, such as 20, 30, and 40 wt.%, and mixing it well mechanically is the most effective way to do so.

Additionally, TiO_2_ is a ceramic substance that may function as an internal pore, so reducing the particle size of the copper. This results in an increase in surface area, which in turn results in an increase in photocatalytic activity. The as-resaved TiO_2_ raw powder amounts in both the anatase and rutile phases and rutile had very minor peaks, which only showed in the high% of TiO_2_ (40%). These findings indicate that the 40% sample contains two different types of tetragonal TiO_2_. However, when the ratio of TiO_2_ is smaller, the rutile peaks do not stand out as clearly because of the low ratio, which causes the peak intensity to be rather low.

Because of the effective milling procedure, Cu and TiO_2_ particles have been found to have achieved a high level of homogeneity across all the samples. When the ball-to-powder ratio is optimized at 20:1, the milling period is extended to 24 h, and the rotating speed is increased to 250 revolutions per minute (rpm), copper particles undergo strain hardening and fracture, which results in a decrease in particle size. The grain size of Cu particles has been reduced following the rise in the percentage of TiO_2_ present. This may be because of the ceramic quality of the TiO_2_ particles; these particles function as internal balls and cause fractures in the Cu particles. For Fig. 4c–f, TiO_2_ particles are well embedded in the Cu particles during the mechanical milling process. Also, they are distributed in the Cu matrix in a good manner. TiO_2_ in nano 50 nm and A Copper powder (supplied by Alpha Chemicals, USA) with a 10 μm. So, the small particles in the SEM images correspond to TiO_2_ and the larger particles belong to copper.

**Figure 5:**
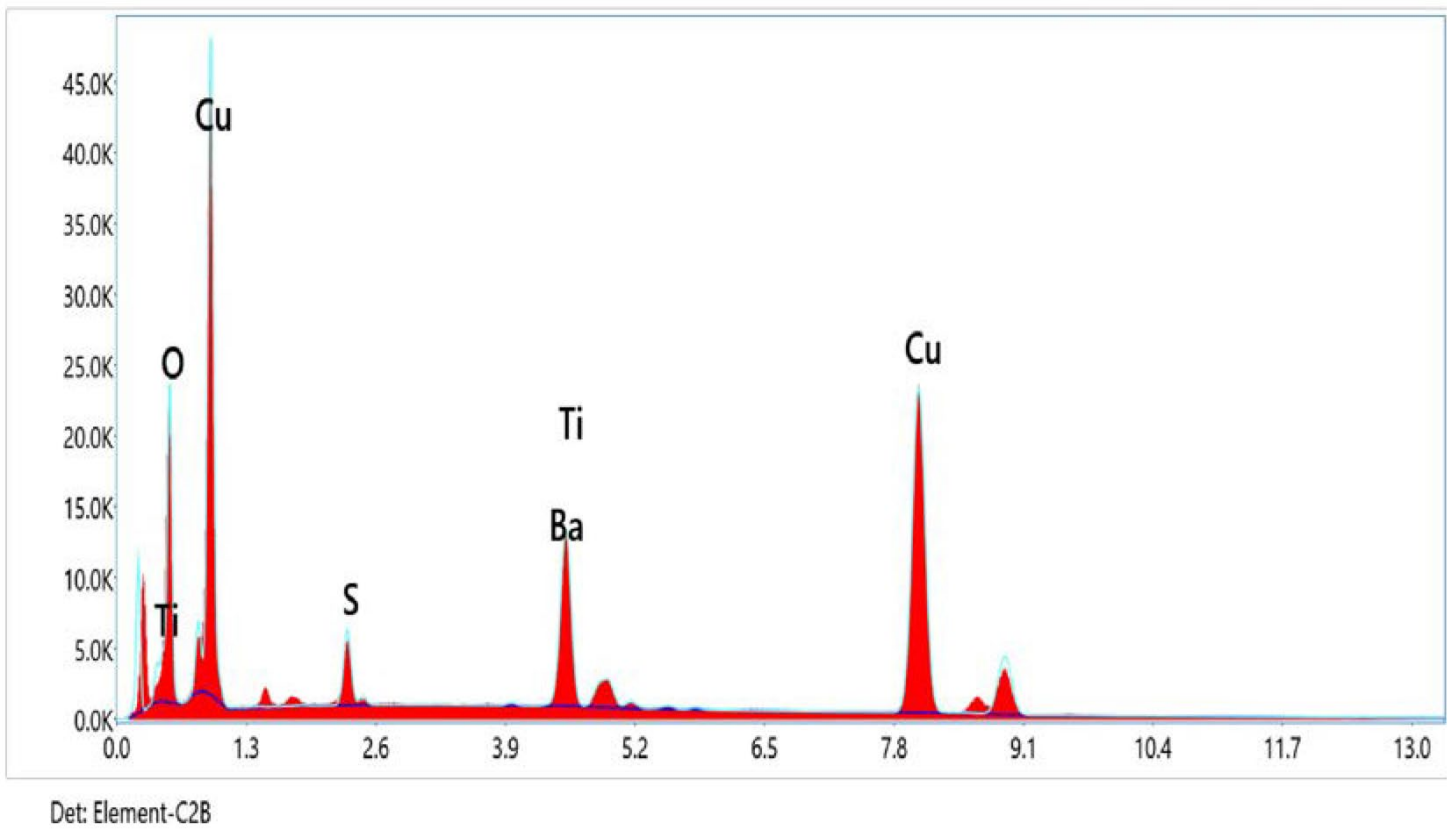
Representative the EDX image of Cu–TiO_2_ nanoparticles and you can see the existence of Cu and Ti with a higher ratio. The blue color represents the Cu without TiO_2_, and the red color represents the Cu with 40 wt.% of TiO_2_. It is noticed that the composite powders contain peaks for Cu, Ti, and O atoms.

Few TiO_2_ particles are aggregates as pockets, for 40wt.% TiO_2_ samples. This may be attributed to the large surface area between the metallic Cu particles and the ceramic TiO_2_ ones. There is no wettability between them. Also, a high difference between their melting points. The EDS analysis of Cu–TiO_2_ samples is shown in Fig. 5 and Table 3 as the composite powders contain peaks for Cu, Ti, and O atoms. And all the prepared composite powders do have not equiaxed grains.

**Table 3:** Representative the EDX data from Fig. 5 of Cu with 40 wt.% of TiO_2_ nanoparticles and you can see the existence of Cu and Ti and O atoms with a higher ratio of 63.84, 8.50, and 18.14 respectively.

Element	Weight %	Atomic %	Net Int	Error %	R	A	F
O K	18.14	46.29	1087.93	9.79	0.8141	0.1423	1.0000
S K	2.69	3.42	406.96	6.07	0.8598	0.5680	1.0149
Ti K	8.50	7.25	1057.11	3.03	0.8886	0.9019	1.0838
Cu K	63.84	41.01	3037.55	2.32	0.9236	0.9681	1.0459
Bal	6.84	2.03	291.69	4.74	0.8932	0.9064	1.0328

### Identification of the bandgap energy

**Figure 6:**
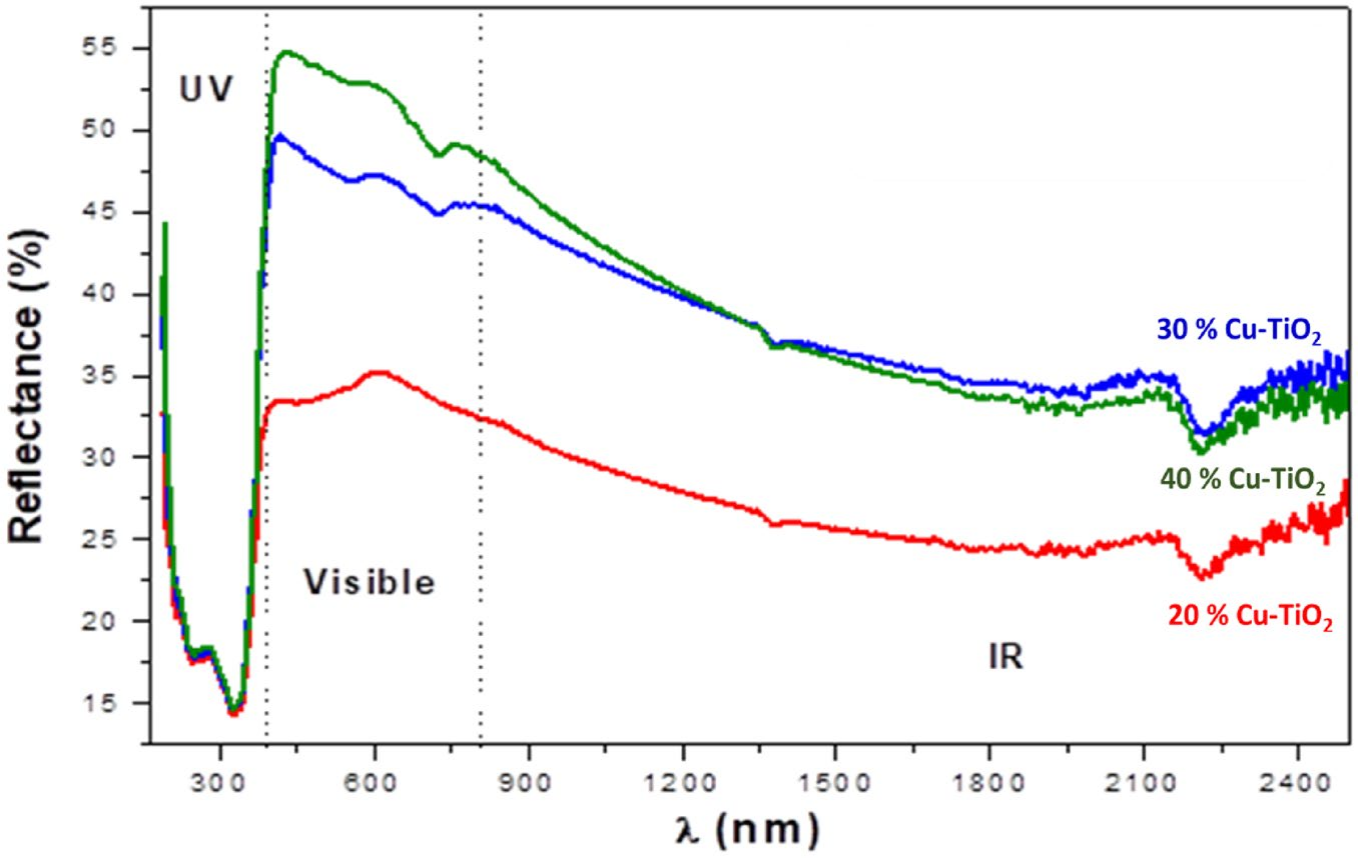
Representative of the UV–vis–IR reflectance spectra of Cu–TiO_2_ with different weight percentages of 20, 30, and 40 wt.% of TiO_2_.

The UV–vis–IR spectra are shown in Fig. 6, and they indicate how the reflectance of the UV–vis–IR spectrum is affected by the different concentrations of produced copper dopant. As the concentration of TiO_2_ increased, it was discovered that the reflectance shifted into the light-visible zone, and this was caused by the shift of the bandgap energy is shown to be lower when there is a higher concentration of TiO_2_. According to the Kubelka–Munk theory, the Schuster–Kubelka–Munk function is given in terms of the optical bandgap (Eg) as:

3 $$\mathrm{F }\left({\mathrm{R}}_{\infty }\right)={\mathrm{ A}\left(\mathrm{hv}-{\mathrm{E}}_{\mathrm{g}}\right)}^{\mathrm{n}},$$

where h is Planck’s constant, ν is the frequency of vibration, and A is a proportionality constant.

**Figure 7:**
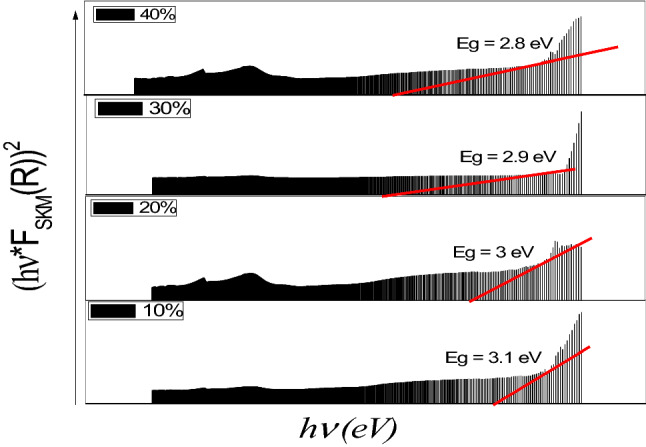
Representative the hvF_SKM_(R))^2^ vs. hv graphs of Cu-TiO_2_ at different weight percent 10, 20, 30, and 40 wt.% of TiO_2_.

The value of the exponent n signifies the nature of the transition, with n = 1/2 or 2 for the direct/indirect allowed transition, respectively. Therefore, the bandgap energy may be evaluated from the reflectance spectra by extrapolating the straight-line plot of (F(R_∞_) *hν)^2^ or (F(R_∞_)*hν)^1/2^ versus (hν) as shown in Fig. 7. Table 4 shows the values of the bandgap for different TiO_2_ concentrations.

**Table 4:** Representative the values of bandgap of Cu–TiO_2_ at different percentages 10, 20, 30, and 40 wt.% of TiO_2_ concentrations.

Cu-TiO_2_	Bandgap in eV
10% Cu–TiO_2_	3.1
20% Cu–TiO_2_	3
30% Cu–TiO_2_	2.9
40% Cu–TiO_2_	2.8

This change in the bandgap can be likely due to the fusion of Ti ions into the Cu crystal structure, and the defect centers formed by the substitution of Cu by Ti ions in the Cu crystal lattice resulting in changes in the optical absorption. The band gap can be determined also by the following formula:

4 $${\mathrm{E}}_{\mathrm{g}}=\frac{\mathrm{hC}}{\uplambda }=1240/{\uplambda }_{\mathrm{cutoff}},$$

where h (Planks constant) = 6.63 × 10^–34^ J.s; C (speed of light) = 3.0 × 10^8^ m/s; λ_cutoff_ (cut off wavelength) = 4.11 × 10^–7^ m. Note: 1 eV = 1.6 × 10^–19^ J (the conversion factor).

The appropriate concentration of TiO_2_ in the Cu matrix is from 20 to 40% for solar cell application. This is because of the necessity of reinforced material in the improvement of light-harvesting of the cell, and the important characteristics of TiO_2_ mesoporous materials. These characteristics like high specific surface area, pore size distribution and providing more reactive sites at surfaces for photocatalytic reactions.

### TiO_2_ antireflection coatings in solar cell

According to the excellent optical properties and low deposition cost of the Titanium dioxide (TiO_2_) thin films, they have a long history in silicon photovoltaics (PV) as antireflection (AR) coatings. This study identifies several unexplored applications for Cu–TiO_2_ thin films, including the enhancement of silicon (Si) solar cell performance, the reduction of costs associated with device manufacture, and the simplification of the preparation process^30^. A technology known as chemical vapor deposition (CVD) was used to deposit Cu–TiO_2_ layers. The facility is provided by the Nano lab at ERI, which aids the team. A single-layer antireflection coating, abbreviated as SLAR, is the bare minimum required for silicon solar cell manufacturing in today’s world. Silicon and other materials that are semiconductors may be used effectively to absorb light. On the other hand, these substances have relatively high refractive indices^31^.

The variety of doping concentrations and shows the spectrum distribution of the optical transmittance of copper doped TiO_2_ films. This figure also demonstrates the range of doping concentrations. The portion of the electromagnetic spectrum that is ultraviolet as well as the visible part were employed to carry out the test that investigated the coated films' level of transmittance. In addition, the optical transmittance values drop when there is a higher concentration of copper. This behavior is brought on by an increase in the number of electrons that are set free whenever there is a greater concentration of copper present in the system.

The refractive index of silicon is n_si_ = 3.939 at 600 nm. This refractive index is much greater than air, which has a constant refractive index of n_0_ = 1.0, and glass (n_0_ = 1.52 at 600 nm). The reflectance of normally incident light at such an interface is given by:

5 $$\mathrm{R}={\frac{({\mathrm{n}}_{\mathrm{si}}-{\mathrm{n}}_{0})}{({\mathrm{n}}_{\mathrm{si}}+{\mathrm{n}}_{0})}}^{2},$$

which means that in the first bounce, about 35.4% or 19.6% of the light is reflected off an air: silicon or glass: silicon interface, respectively. If an optimum-thickness AR coating is inserted between the silicon and ambient medium, the minimum reflectance is given by:

6 $$\mathrm{R}={(\frac{{\mathrm{n}}_{\mathrm{AR}}^{2}-{\mathrm{n}}_{0}{\mathrm{n}}_{\mathrm{si}}}{{\mathrm{n}}_{\mathrm{AR}}^{2}+{\mathrm{n}}_{0}{\mathrm{n}}_{\mathrm{si}}})}^{2},$$

where n_AR_ is the coating refractive index. To achieve zero reflectance at one wavelength, the value of n_AR_ should be.

7 $${\mathrm{n}}_{\mathrm{AR}}=\sqrt{{\mathrm{n}}_{0}{\mathrm{n}}_{\mathrm{si}}},$$

and the film thickness (d_AR_) must meet the quarter-wave optical thickness requirement which can be formulated as:

8 $${\mathrm{d}}_{\mathrm{AR}}=\frac{{\uplambda }_{0}}{4{\mathrm{n}}_{\mathrm{AR}}},$$

The formula is related to the design of antireflection coatings for optical surfaces. Here are the relevant bases:d_AR_ represents the thickness of the antireflection coating in nanometers (nm), λ_0_ represents the wavelength of the incident light in vacuum, typically in units of nanometers (nm), n_AR_ represents the refractive index of the antireflection coating at the wavelength λ_0_.

The formula is derived from the principle of optical interference. When light is incident on a thin film with a thickness d and refractive index n, some of the light is reflected at the air-film interface, and some of it is transmitted through the film. The reflected and transmitted light waves interfere with each other, and the resulting interference pattern determines the amount of reflected light. For an antireflection coating, the goal is to minimize the amount of reflected light at a specific wavelength λ_0_. This can be achieved by choosing a thickness d_AR_ and refractive index n_AR_ such that the reflected light waves interfere destructively, cancelling each other out. The formula d_AR_ = λ_0_/(4n_AR_) gives the optimal thickness of the antireflection coating for achieving this interference pattern at the wavelength λ_0_.

**Figure 8:**
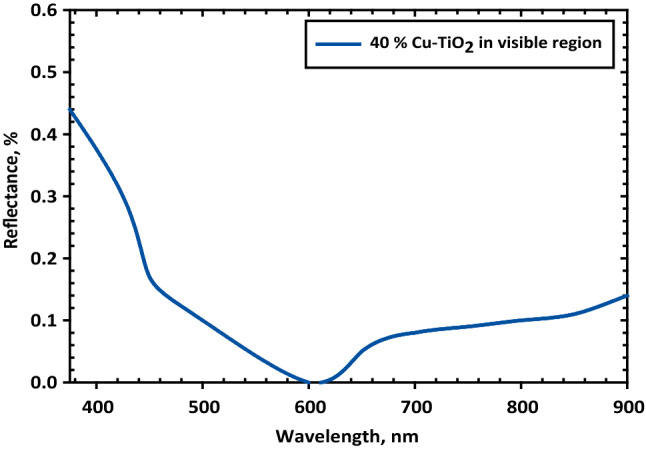
Representative the Cu–TiO_2_ as an AR coating for a silicon solar cell.

There are many parameters for choosing the antireflection material like resist corrosion, withstand high temperatures, and other many parameters. Cu reinforced with TiO_2_ can be used as the optimum material for SLAR. Controlling the ratio of TiO_2_ in copper can achieve the required film thickness and reflectivity. Due to Eqs. (5) and (6), The AR coating should have 1.98 and 75.6 nm for refractive index and thickness, respectively. These values can achieve by using a Cu–TiO_2_ composite. Figure 8 showed the Cu–TiO_2_ as an AR coating for a silicon solar cell. It can be achieved under the condition of a fixed reflection index at the visible region^32^.

### Electrical and thermal conductivities

**Figure 9:**
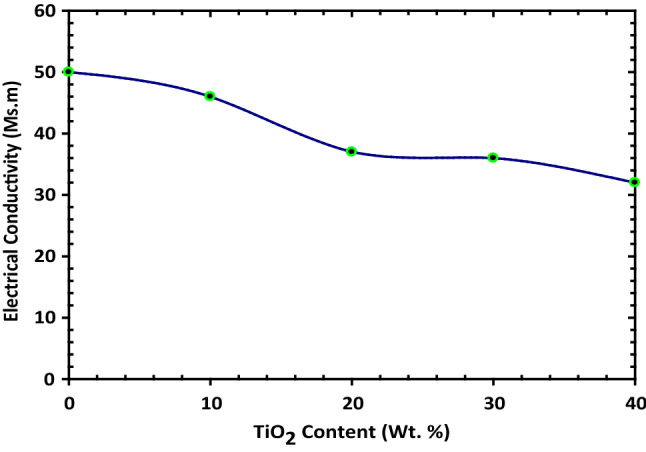
Representative the electrical conductivity of Cu–TiO_2_ at different percentages 10, 20, 30, and 40 wt.% of TiO_2_ Content.

A solar cell is an electrical device that converts light energy directly by the photovoltaic effect. It is a type of photoelectric cell. So, it has electrical characteristics, like current, electrical resistance, or voltage which vary when exposed to light. Solar cell considers electrical building blocks of photovoltaic modules, called solar panels. Electrons are excited from their orbital. It can dissipate the energy as heat and returns to its orbital. Current flows through the material to cancel the potential and this electricity is captured. So, studying the electrical and thermal conductivity is a good indication of the quality of the solar cell. Figure 9 shows the effect of TiO_2_ additions on the electrical conductivity of Cu-TiO_2_ nanocomposite powders. It decreases gradually by increasing the TiO_2_%. This can be attributed to the lower electrical conductivity of TiO_2_ than that of Cu. As the electrical resistivity of TiO_2_ is 420 nΩ.m, while that of Cu is 16.78 nΩ.m. So, TiO_2_ resists the following of electronic charge carriers more than Cu. Consequently, the electrical conductivity decreases^33^.

**Figure 10:**
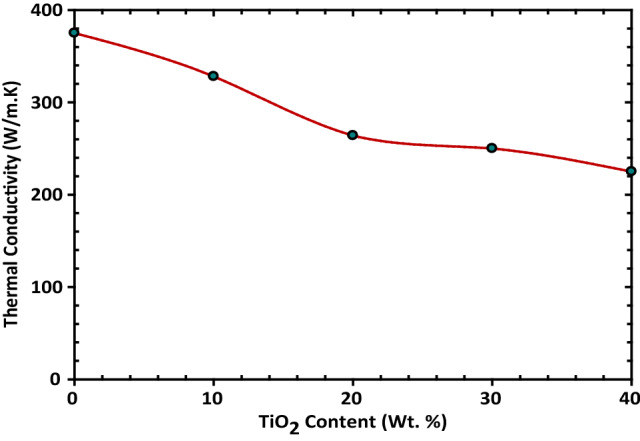
Representative the thermal conductivity of Cu–TiO_2_ at different percentages 10, 20, 30, and 40 wt.% of TiO_2_ content.

Figure 10 displays the relation between the TiO_2_% and the thermal conductivity of the Cu matrix. It is decreased gradually by increasing the TiO_2_%. This can be explained by the lower thermal conductivity value of TiO_2_ than that of Cu, which, is 21.9 W/m.K for TiO_2_ & 401 W/m.K for Cu. So, according to the rule of mixture the overall thermal conductivity of Cu-TiO_2_, nanocomposites is decreased by the addition of lower conductivity TiO_2_ particles. In which TiO_2_ restricts the heat transfer in the Cu matrix.

It must be noted that, although TiO_2_ additions to the copper matrix, decreases both the electrical and thermal conductivities, it is still in the working area of Cu applications. As reinforcing Cu with TiO_2_ did not convert copper into non-conductive material, only decreases its conductivity.

## Conclusions

In this paper, Cu–TiO_2_ nanocomposite powders have been successfully prepared by the mechanical milling method. In this preparation method, the weighted composite powders were mixed in a stainless-steel vial and protected from oxidation using pure argon, by a 20:1 steel ball-to-powder ratio (BPR), a ball diameter of 5 mm, and a rotational speed of 250 rpm. Various content of nano-TiO_2_ particles successfully reinforced the Cu matrix composite and uniformly distributed inside the matrix through the fabrication process of the powder metallurgy technique. Cu–TiO_2_ has been characterized by using Fourier transform infrared spectroscopy (FTIR), X-ray diffraction (XRD), Scanning Electron Microscope (SEM) to determine their crystal structure, and UV–visible absorption spectrometry (UV–Vis) to estimate the optical properties. The X-ray diffraction pattern showed peaks corresponding to Cu and TiO_2_. There was no record of any other interfering intermetallic compounds in the XRD pattern. On the other hand, SEM images showed a proper and homogenous dispersal of TiO_2_ in the fabricated composite matrix. This paper also studied the influence of various prepared TiO_2_ dopant concentrations on the UV–vis-IR reflectance. It can be observed that increasing TiO_2_ concentration, increases the reflection % which is good in different applications related to solar cell fabrication.

## Data Availability

All data generated or analyzed during this study are included in this published article.
